# Secondhand smoke exposure and asthma status among adolescents: Findings from the 2019–2020 California Student Tobacco Survey

**DOI:** 10.1016/j.pmedr.2024.102842

**Published:** 2024-07-27

**Authors:** Nora Satybaldiyeva, Anthony Gamst, Eyal Oren, Ji-Yeun Park, Shu-Hong Zhu

**Affiliations:** a Herbert Wertheim School of Public Health, University of California San Diego, La Jolla, CA, USA; b School of Public Health, San Diego State University, San Diego, CA, USA; c Department of Mathematics, University of California San Diego, La Jolla, CA, USA; d Department of Kinesiology and Public Health, Biola University, La Mirada, CA, USA; e Moores Cancer Center, University of California San Diego, La Jolla, CA, USA

**Keywords:** Asthma, Secondhand smoke, Cannabis, Marijuana, Tobacco

## Abstract

**Background and objectives:**

Secondhand tobacco smoke is associated with worsening asthma symptoms among children. However, the relationship between secondhand marijuana smoke and asthma symptoms among youth has not been examined. This study compares the prevalence of secondhand tobacco and marijuana smoke exposure, overall and by asthma status, among middle and high school students.

**Methods:**

The study assessed participants of the 2019–2020 California Student Tobacco Survey: a large, cross-sectional random sample of 8th, 10th, and 12th graders (N = 158,937). Descriptive analyses examined exposure to combustible tobacco and marijuana secondhand smoke by students’ asthma status and sociodemographic characteristics.

**Results:**

More students with asthma were exposed to combustible tobacco secondhand smoke (13.4 %) and marijuana secondhand smoke (12.0 %) than students without asthma (10.9 % and 9.3 %, respectively). The prevalence of secondhand marijuana smoke exposure was higher among 12th grade students (12.2 %) while the prevalence of secondhand tobacco smoke exposure was higher among 8th grade students (13.4 %) and those who lived in rural locations (15.4 %).

**Conclusions:**

California students are exposed to marijuana secondhand smoke at similar proportions to combustible tobacco secondhand smoke and more students with asthma are exposed to marijuana secondhand smoke. These results expand the public health issue of secondhand smoke exposure among children with asthma by highlighting the need to examine marijuana secondhand smoke. Given the rapid shift in marijuana laws across the US, exposure to secondhand marijuana smoke is likely to increase. Therefore, vulnerable populations, such as children with asthma, should be prioritized for interventions that aim to alleviate secondhand marijuana exposure.

## 1 Introduction

Asthma is the most common chronic condition in children in the United States (US), with over 4.5 million youth affected (Prevention, 2021). Many of the US children with asthma (38.7 %) suffered an asthma attack in 2021 (Prevention, 2021). Children with asthma are more sensitive to certain environmental triggers, such as pollutants and particles in the air (Agency and Assessment, 2005, Prevention, C.f.D.C.a., 2021). Additionally, secondhand tobacco smoke has been causally associated with worsening asthma symptoms among up to 1 million children (Agency and Assessment, 2005). A systematic review of 93 studies showed that secondhand smoke is associated with the development of doctor-diagnosed asthma, wheezing, and asthma-like syndrome in childhood (He et al., 2020). Childhood is a critical time for examining the association between secondhand smoke and asthma prevalence as it is the time when asthma often begins, assessment of secondhand smoke exposure is more straightforward, and the prevalence of smoking among children is low (He et al., 2020).

While there is evidence that secondhand tobacco smoke exposure in adolescence is an ongoing public health issue, the prevalence of marijuana secondhand smoke among children and its effects on health are unknown. There are documented reports of cannabis allergy in children which suggest that secondhand marijuana smoke exposure may contribute to asthma in young children (Hoffman et al., 2020). Prior research has also shown positive associations between secondhand marijuana smoke and viral respiratory infections in children (Johnson et al., 2021). Another study has shown that indoor marijuana smoke was positively associated with a greater number of adverse health outcomes, such as emergency department use for coughing, physician-diagnosed bronchitis, and physician-diagnosed asthma, even after adjusting for exposure to tobacco smoke (Posis et al., 2019). Lastly, asthma prevalence among youth has increased by over 2.5-fold among states that have passed recreational marijuana laws compared to states where marijuana is fully illegal (Goodwin et al., 2023). Therefore, it is important to understand the proportion of youth exposed to secondhand marijuana smoke, their current asthma status, and how they compare to youth exposed to secondhand tobacco smoke.

Herein, we aimed to estimate the prevalence of secondhand marijuana smoke and its association with asthma status in a large, random sample of adolescents. As a secondary objective, we compared the prevalence of secondhand marijuana smoke to the prevalence of secondhand tobacco smoke in the same sample. Finally, we examined the demographic characteristics of students who were exposed to secondhand tobacco and marijuana smoke to note any meaningful differences.

## 2 Methods

The study sample consisted of students who participated in the 2019–2020 California Student Tobacco Survey (CSTS), which was the largest wave of the survey that collected information related to asthma and secondhand smoke exposure to tobacco and marijuana. The CSTS is a cross-sectional, state-representative survey funded by the California Department of Public Health that assesses the use of and perceptions towards tobacco, marijuana, and other drugs among youth. As described elsewhere (Zhu, 2021), the CSTS uses a two-stage cluster sampling design in which the school is the primary sampling unit and classroom is the secondary sampling unit. The survey was administered online by the University of California, San Diego to randomly selected middle and high schools throughout California from September 2019 to March 2020 before the COVID-19 school closures. Within each school, only 8th, 10th, and 12th graders were sampled. The 2019–2020 CSTS approached a random sample of 608 schools, and 482 agreed to participate (79.3 %). Among these, a total of 358 schools (79.3 %) and 162,675 students completed the survey, with an overall student response rate of 68.3 %. This study focuses on students who responded to questions about asthma and exposure to secondhand marijuana smoke; thus 3738 students who did not respond to those questions were excluded from analysis. The effective sample consisted of 158,937 students across California. This study was approved by the University of California San Diego Human Research Protections Program (#170787), the California State Committee for the Protection of Human Subjects (#15–04-1992), and appropriate school district committees.

Asthma status was assessed using a question that asked, “Has a doctor or nurse ever told you that you have asthma?” Students could answer “yes”, “no”, or “I don’t know”. Students who answered “yes” or “I don’t know” were then asked whether they had an asthma attack in the past 12 months.

Exposure to secondhand marijuana smoke was measured using two questions which asked whether they were in a i) room or ii) car when someone was smoking marijuana in the last two weeks. Similarly, exposure to secondhand combustible tobacco smoke asked whether students were in a i) room or ii) car when someone was smoking a cigarette, little cigar, or cigarillo in the last two weeks. Students answered each question as “yes” or “no”. For the purposes of this analysis, we combined those who responded “yes” to at least one of the two questions into a category that we defined as being exposed to secondhand smoke either in a room or car.

The survey also collected demographic information on characteristics associated with asthma indicators (Pate, 2021), which included the student’s race/ethnicity, gender (male, female, or other), grade (8th, 10th, or 12th), and location (rural or urban). Current users of marijuana were students who reported using a marijuana product in the last 30 days. Non-current users of marijuana were those who reported never using marijuana (never users) and those who reported ever using marijuana but not in the last 30 days (former users). Similarly, the non-current combustible tobacco user category included never users and former users of cigarettes, little cigars, and cigarillos. This study was subset to non-current users of tobacco and marijuana to prevent bias that may arise from smoke produced by the respondent’s own use.

We first calculated the proportions of students with asthma and asthma attack in the past year who were exposed to secondhand marijuana and combustible tobacco smoke in a room, in a car, or either in a room or in a car among current users of marijuana and cigarettes. Afterwards, we examined the prevalence of secondhand marijuana smoke exposure and secondhand combustible tobacco smoke exposure by each of the demographic characteristics. We used survey weights to produce population-based estimates. Weights were calibrated to the state grade distribution so that the total of the survey weights by grade would be equivalent to the actual enrollment in each grade. Finally, we constructed two multivariable logistic regression models to examine 1) the association between secondhand marijuana and asthma status and 2) the association between combustible tobacco exposure and asthma status, while controlling for individual demographic characteristics. All data management and analyses were performed using SAS, version 9.4.

## 3 Results

Among non-current users of marijuana, students with asthma were exposed to marijuana secondhand smoke either in a room or car (12.0 %) at higher proportions than those without asthma (9.3 %) (Table 1). Similarly, students with an asthma attack in the past year were exposed to marijuana secondhand smoke either in a room or car (16.5 %) at much higher proportions than those without an asthma attack (10.0 %). The differences were similar, although much smaller, among non-current users of combustible tobacco who reported exposure to secondhand combustible tobacco smoke. Students with asthma and a past year asthma attack were exposed to combustible tobacco secondhand smoke in a room or car (13.4 % and 19.6 %, respectively) at higher proportions than those without asthma (10.9 %) and those without an asthma attack (16.0 %).

**Table 1:** Differences in exposure to secondhand smoke, among non-current users of marijuana and combustible tobacco, respectively, California Student Tobacco Survey, 2019–2020 (N = 158,937).

**Non-Current Marijuana Users**	**n**	**Marijuana Secondhand Smoke Exposure**		
		**In a Room** *% (95 % CI)*	**In a Car** *% (95 % CI)*	**Either Room or Car** *% (95 % CI)*
**Asthma**				
Yes	24,934	10.4 (9.7–11.1)	5.5 (4.9–6.0)	12.0 (11.3–12.8)
No	99,765	7.8 (7.3–8.3)	3.9 (3.7–4.2)	9.3 (8.8–9.9)
I don’t know	12,366	6.9 (6.0–7.8)	4.8 (4.2–5.5)	8.9 (7.8–9.9)

**Asthma Attack**				
Yes	4860	14.3 (12.5–16.1)	7.9 (6.3–9.5)	16.5 (14.8–18.2)
No	26,996	8.3 (7.6–9.1)	4.6 (4.1–5.2)	10.0 (9.1–10.9)
I don’t know	5372	8.1 (6.7–9.5)	5.6 (4.5–6.7)	9.8 (8.3–11.3)

**Non-Current Combustible Tobacco Users**	**n**	**Combustible Tobacco Secondhand Smoke Exposure**		

**In a Room** *% (95 % CI)*	**In a Car** *% (95 % CI)*	**Either Room or Car** *% (95 % CI)*

**Asthma**				
Yes	28,499	10.0 (9.2–10.7)	7.8 (7.0–8.7)	13.4 (12.4–14.4)
No	112,282	7.8 (7.4–8.3)	5.8 (5.4–6.3)	10.9 (10.3–11.4)
I don’t know	13,937	9.8 (8.3–11.2)	9.5 (8.3–10.8)	14.4 (12.8–16.1)

**Asthma Attack**				
Yes	5589	14.1 (12.4–15.8)	11.5 (9.9–13.1)	19.6 (16.9–21.1)
No	30,526	8.8 (7.9–9.9)	7.2 (6.4–8.0)	12.3 (11.2–13.4)
I don’t know	6236	11.1 (9.4–12.9)	11.6 (9.8–13.4)	16.0 (14.1–18.0)

Secondhand marijuana smoke was significantly associated with having asthma in both unadjusted and fully adjusted models among adolescent non-current users of marijuana. After adjusting for grade, gender, race/ethnicity, urbanicity, and secondhand combustible tobacco smoke, youth exposed to marijuana secondhand smoke were 23 % more likely to have asthma than adolescents without secondhand marijuana smoke exposure (aOR = 1.23, 95 % CI: 1.16, 1.33). There was no association between secondhand combustible tobacco smoke exposure and asthma status in both unadjusted and fully adjusted models among adolescent non-current users of combustible tobacco (aOR = 1.01, 95 % CI: 0.92, 1.10). The results of our fully adjusted models are presented in Supplemental Table 1.

The demographic characteristics with respect to secondhand marijuana and combustible tobacco smoke exposure among California students are presented in Table 2. The prevalence of secondhand marijuana smoke exposure was lowest among students who identified as Non-Hispanic Asian (4.5 %) and highest among students who identified as Non-Hispanic Black (13.9 %). The prevalence of secondhand combustible tobacco smoke exposure was lowest among students who identified as Hispanic (10.3 %) and highest among students who identified as Non-Hispanic American Indian/Alaskan Native (19.5 %). The prevalence of secondhand marijuana and combustible tobacco smoke exposure was highest among students who identified their gender as other (14.5 % and 19.0 %, respectively). The prevalence of secondhand marijuana smoke exposure was highest among 12th grade students (12.2 %) while the prevalence of secondhand combustible tobacco smoke exposure was highest among 8th grade students (13.4 %). While the prevalence of secondhand combustible tobacco smoke exposure was higher among students living in rural areas than those living in urban areas (15.4 % vs 11.1 %), secondhand marijuana smoke exposure did not differ by geographic location.

**Table 2:** Demographic characteristics of students exposed to marijuana and combustible tobacco secondhand smoke, California Student Tobacco Survey, 2019–2020 (N = 158,937).

	**Non-Current Marijuana Users**	**Marijuana Secondhand Smoke Exposure**	**Non-Current Combustible Tobacco Users**	**Combustible Tobacco Secondhand Smoke Exposure**
	**n** = 137,063	*% (95 % CI)*	**n** = 154,718	*% (95 % CI)*
**Race/Ethnicity**				
NH-White	35,644	12.4 (11.0–13.7)	40,915	14.3 (12.9–15.7)
NH-Black	4535	13.9 (11.8–15.9)	5241	15.5 (13.4–17.7)
Hispanic	71,344	9.6 (9.1–10.1)	81,137	10.3 (9.7–10.8)
NH-Asian	20,269	4.5 (3.9–5.1)	21,417	10.7 (9.8–11.5)
NH-AI/AN	607	11.0 (6.5–15.6)	730	19.5 (12.8–26.2)
NH-NHOPI	912	8.8 (6.0–11.5)	1084	14.9 (11.3–18.4)
NH-Other	3296	7.4 (6.1–8.7)	3661	13.9 (11.5–16.3)
*Missing*	456		533	

**Gender**				
Male	63,233	9.2 (8.6–9.7)	70,490	10.4 (9.7–11.0)
Female	67,017	9.8 (9.2–10.5)	76,194	12.3 (11.6–12.9)
Other*	6517	14.5 (12.1–16.8)	7683	19.0 (17.3–20.8)
*Missing*	296		351	

**Grade**				
8	11,127	7.2 (6.0–8.4)	11,584	13.4 (12.0–14.8)
10	70,389	10.3 (9.8–10.9)	77,976	11.3 (10.7–12.0)
12	55,547	12.2 (11.6–12.8)	65,158	10.3 (9.9–10.8)

**Location**				
Rural	19,057	10.2 (8.0–12.4)	21,912	15.4 (13.2–17.6)
Urban	118,006	9.7 (9.2–10.2)	132,806	11.1 (10.6–11.5)

Abbreviations: NH = Non-Hispanic; AI = American Indian; AN = Alaskan Native; NHOPI = Native Hawaiian or Other Pacific Islander.

* Other gender includes transgender, genderqueer, and other gender.

## 4 Discussion

In this large, representative sample of California students, we found that more adolescents with asthma were exposed to marijuana secondhand smoke and combustible tobacco secondhand smoke than adolescents without asthma. While the rates of combustible tobacco secondhand smoke exposure were slightly higher, the difference was not statistically significant, which raises concern that children in California are exposed to marijuana secondhand smoke at similar rates to tobacco secondhand smoke. This is especially concerning as our results show that there is an association between secondhand marijuana smoke exposure and asthma status.

While demographic differences were not the focus of the current research, we found that more older students were exposed to secondhand marijuana smoke while more younger students were exposed to secondhand tobacco smoke. One possible explanation for this finding is that 12th grade students may be spending more time with peers, who use marijuana at higher rates than combustible tobacco, while 8th grade students may be spending more time with adults/caregivers, who are more likely to use combustible tobacco (Simons-Morton and Chen, 2006). Additionally, a higher percentage of students living in rural settings were exposed to secondhand tobacco smoke; potentially as a result of higher tobacco smoking rates in rural counties throughout California (California Department of Public Health, 2022).

It is important to note that the cross-sectional design of the current study does not allow us to conclude whether there is a causal effect between exposure to secondhand tobacco and marijuana smoke and asthma among adolescents. Additionally, students self-reported their asthma status, which could have led to a less accurate measure of the disease than a physician-confirmed diagnosis. There may be residual confounding by factors not assessed in the CSTS, such as socioeconomic status. Despite the limitations, however, this is the first study to date that provides the prevalence of secondhand marijuana smoke exposure among youth and shows an association between secondhand marijuana smoke exposure and the presence of asthma, as well as asthma attacks. A major strength of this study is the large, representative sample of 8th, 10th, and 12th grade students across California. The timeframe of the current survey provides an up-to-date snapshot of the current prevalence of secondhand tobacco and marijuana smoke in California. To our knowledge, this is the first-of-a-kind analysis comparing secondhand marijuana smoke and combustible tobacco smoke exposure and asthma status among youth.

### 4.1 Conclusion

Our findings further expand the public health issue of secondhand smoke exposure among children with asthma by highlighting the need to examine marijuana secondhand smoke in addition to tobacco smoke. Given the rapid, historic shift in policies around medical and recreational marijuana use in the US over the last decade (Project, 2021), the general population's exposure to secondhand marijuana smoke is likely to increase. Already, asthma rates have been shown to be higher in youth residing in states with more permissible marijuana laws (Goodwin et al., 2023). Therefore, vulnerable populations, such as children with asthma, should be prioritized for interventions that aim to alleviate secondhand marijuana exposure.

## Funding/Support

This study was supported by a contract from the California Department of Public Health #CDPH-16-10109.

## Role of Funder/Sponsor (if any)

The study funders did not play any role in the design and conduct of the study.

## CRediT authorship contribution statement

**Nora Satybaldiyeva:** Writing – original draft, Investigation, Formal analysis, Conceptualization. **Anthony Gamst:** Writing – review & editing, Methodology, Investigation. **Eyal Oren:** Writing – review & editing, Investigation. **Ji-Yeun Park:** Writing – review & editing, Investigation. **Shu-Hong Zhu:** Writing – review & editing, Methodology, Investigation, Funding acquisition, Conceptualization.

## Declaration of competing interest

The authors declare that they have no known competing financial interests or personal relationships that could have appeared to influence the work reported in this paper.

## Data Availability

Data will be made available on request.
