# Supplementary material for: Secondhand smoke exposure and asthma status among adolescents: Findings from the 2019–2020 California Student Tobacco Survey
Source: Prev Med Rep. 2024 Jul 27;45:102842. doi: 10.1016/j.pmedr.2024.102842 (PMC11342744; doi:10.1016/j.pmedr.2024.102842)
Supplement: Supplementary Data 1 [file mmc1.docx]

**Supplemental Table 1**. Adjusted Measures of Association Between Asthma Status and Secondhand Combustible Tobacco and Marijuana Smoke Exposure; California Student Tobacco Survey, 2019-2020.

|  | **Combustible tobacco Adjusted OR (95% CI)** | **Marijuana**  **Adjusted OR (95% CI)** |
| --- | --- | --- |
|  |  |  |
| **Combustible tobacco secondhand smoke** |  |  |
| Unexposed | Ref | Ref |
| Exposed | 1.01 (0.92, 1.10) | 1.01 (0.92, 1.10) |
| **Marijuana secondhand smoke** |  |  |
| Unexposed | Ref | Ref |
| Exposed | 1.21 (1.13, 1.30) | 1.23 (1.16, 1.33) |
| **Race/**  **Ethnicity** |  |  |
| NH-White | Ref | Ref |
| NH-Black | 1.12 (0.96, 1.30) | 1.10 (0.93, 1.31) |
| Hispanic | 0.87 (0.82, 0.92) | 0.85 (0.81, 0.90) |
| NH-Asian | 0.89 (0.84, 0.95) | 0.88 (0.82, 0.94) |
| NH-AI/AN | 0.54 (0.39, 0.76) | 0.54 (0.38, 0.79) |
| NH-NHOPI | 0.77 (0.57, 1.05) | 0.83 (0.59, 1.16) |
| NH-Other | 0.55 (0.46, 0.66) | 0.52 (0.43, 0.62) |
| **Gender** |  |  |
| Male | Ref | Ref |
| Female | 0.95 (0.90, 0.99) | 0.94 (0.90, 0.99) |
| Other* | 0.42 (0.37, 0.49) | 0.43 (0.36, 0.50) |
| **Grade** |  |  |
| 8 | Ref | Ref |
| 10 | 1.19 (1.09, 1.29) | 1.18 (1.09, 1.28) |
| 12 | 1.30 (1.20, 1.41) | 1.31 (1.20, 1.42) |
| **Location** |  |  |
| Rural | Ref | Ref |
| Urban | 1.04 (0.96, 1.14) | 1.01 (0.92, 1.10) |

Abbreviations: OR = odds ratio; NH = Non-Hispanic; AI = American Indian; AN = Alaskan Native; NHOPI = Native Hawaiian or Other Pacific Islander

*Other gender includes transgender, genderqueer, and other gender.
